# Immunostimulatory Activity of *Lactococcus lactis* subsp. *lactis* CAB701 Isolated from Jeju Cabbage

**DOI:** 10.3390/microorganisms11071718

**Published:** 2023-06-30

**Authors:** Huijin Jeong, Suin Kim, Un-Sik Hwang, Hyukjoon Choi, Young-Seo Park

**Affiliations:** 1 Department of Food Science and Biotechnology, Gachon University, Seongnam-si 13120, Republic of Korea; huijin0218@gmail.com (H.J.);; 2 BKbio Co., Ltd., Jeju-si 63359, Republic of Korea

**Keywords:** probiotics, lactic acid bacteria, *Lactococcus lactis*, immunostimulatory activity, nitric oxide production, mitogen-activated protein kinase pathway, gastrointestinal tolerance, antioxidant activity

## Abstract

This study explored the potential of *Lactococcus lactis* subsp. *lactis* CAB701 as a probiotic strain, focusing on its immunostimulatory properties. Despite adverse conditions in the gastrointestinal environment, this strain exhibited remarkable survivability, as evidenced by its tolerance to acid, bile, and pancreatin, coupled with its impressive ability to adhere to Caco-2 cells. It also exhibited significant antioxidant activity, similar to the established probiotic *Lacticaseibacillus rhamnosus* GG (LGG). Our research elucidates the potent immunostimulatory effects of *L. lactis* subsp. *lactis* CAB701. This strain significantly enhanced nitric oxide production in RAW 264.7, far exceeding that obtained with LGG. An in-depth examination revealed elevated expression of key inflammatory mediators, including inducible nitric oxide synthase, tumor necrosis factor-alpha, cyclooxygenase-2, interleukin (IL)-1 beta, and IL-6. *L. lactis* subsp. *lactis* CAB701 increases the expression of critical signaling proteins in the mitogen-activated protein kinase pathway. This prompted a substantial increase in the expression of phosphorylated c-Jun *N*-terminal kinases and extracellular signal-regulated kinases, suggesting their role in modulating these immune-related pathways. Overall, these findings demonstrate the significant immunostimulatory capacity of *L. lactis* subsp. *lactis* CAB701, positioning it as a potential candidate for probiotic use, especially in applications that enhance immune responses.

## 1. Introduction

Probiotics, defined as “live microorganisms that, when administered in adequate amounts, confer a health benefit to the host” [1], have become an essential component of modern health science and dietetics owing to their potential health benefits. Extensive research has underscored the importance of maintaining a healthy gut microbiota, which is vital for overall wellbeing. One of the primary roles of probiotics is to restore and maintain a balanced gut microbiota [2]. Disruptions to the gut microbiota, whether caused by diet, illness, or medications such as antibiotics, can lead to dysbiosis, a condition associated with numerous health problems, including digestive disorders, allergies, and mental health conditions. By introducing beneficial bacteria into the gastrointestinal (GI) tract, probiotics can help restore the gut microbiota balance and promote digestion and nutrient absorption [3]. Probiotics have also been observed to enhance immune responses. Probiotic bacteria stimulate the body’s natural defense mechanisms, boost the activity of immune cells, and promote antibody production [4]. Immune modulation can help the body respond more effectively to infections, reducing infectious disease incidence and severity [5]. Probiotics may also improve mental health. Recent studies have indicated a connection between the gut microbiota and the brain, called the gut-brain axis. Probiotics have been suggested to influence brain function and mental health through this axis, with potential benefits for conditions such as depression and anxiety [6]. Certain probiotics are associated with weight management and may play a role in combating obesity [7]. They may influence metabolic processes and potentially contribute to weight loss and management. Given these promising health benefits, the study and application of probiotics hold significant potential for improving human health and treating various health conditions.

*Lactococcus lactis*, a lactic acid bacterium, has long been used to manufacture dairy products, such as cheese and buttermilk. Its historical use in food fermentation and safety for human consumption has led to its status as a Generally Recognized as Safe (GRAS) organism [8]. Recently, *L. lactis* has gained considerable attention as a promising probiotic because of its potential health benefits. *L. lactis* is a facultatively anaerobic, Gram-positive bacterium known for its ability to survive the harsh conditions of the human GI tract. It can resist low pH levels in the stomach and tolerate bile salts in the intestine [9]. This survival capability enables it to reach the gut in an active state, which is a vital feature for probiotics. In addition to its survival traits, *L. lactis* shows promising immunomodulatory capabilities. It can stimulate cytokine production, enhance macrophage activity, and modulate immune and inflammatory signaling [10]. Its ability to modulate immune responses has potential therapeutic applications in immune-related disorders, such as inflammatory bowel disease and allergies.

*L. lactis* possesses a broad range of immunomodulatory capabilities, including enhancing phagocytic cell activity, promoting proinflammatory cytokine production, and modulating immune and inflammatory signaling pathways. Proinflammatory cytokines are chemical messengers that play crucial roles in regulating immune responses and inflammation. *L. lactis* stimulates the production of cytokines such as interferon-γ and tumor necrosis factor (TNF)-α via immune cells, strengthening the defenses of the body against infectious agents [11,12]. Moreover, *L. lactis* modulates various immune and inflammatory signaling pathways, including the nuclear factor-κB (NF-κB) and mitogen-activated protein kinase (MAPK) pathways, which are essential for initiating and regulating immune responses. *L. lactis* maintains immune homeostasis and prevents excessive or inappropriate immune reactions that can harm the host by manipulating these pathways. Collectively, these immunomodulatory properties make *L. lactis* a promising candidate for developing probiotic therapies that target a range of immune-related disorders, including inflammatory bowel disease, allergies, and autoimmune conditions.

This study investigated the survival mechanisms of *L. lactis* subsp. *lactis* CAB701 in the human GI tract. Its acid tolerance, bile resistance, and resilience against pancreatin were examined in detail, as these factors are key determinants of the capacity of a probiotic to effectively reach the gut, maintain its viability, and subsequently confer health benefits. Subsequently, the immunomodulatory potential of *L. lactis* subsp. *lactis* CAB701 was analyzed. Building on previous studies highlighting the ability of *L. lactis* to stimulate proinflammatory cytokine production and modulate immune and inflammatory signaling, we assessed whether these inherent properties were present in *L. lactis* subsp. *lactis* CAB701. This may provide a broader understanding of its therapeutic potential. The findings of this study significantly enhance our understanding of the role of probiotics in immune health. Most importantly, *L. lactis* subsp. *lactis* CAB701 demonstrating promising results can pave the way for its use as a novel probiotic to promote gut health or as a potential therapeutic agent for treating immune-related disorders.

## 2. Materials and Methods

### 2.1. Isolation of Lactic Acid Bacteria Strains and Growth Conditions

To isolate lactic acid bacteria, eight types of local plants from Jeju Island, namely carrots, cactus, broccoli, beets, cabbage, hallabong, kale, and kohlrabi, were obtained from BKbio Co., Ltd. (Jeju, Republic of Korea). The plants were appropriately cut and placed in stomacher bags containing sterile 0.85% (*w*/*v*) NaCl. The samples were homogenized using a stomacher homogenizer (BNF Korea, Gimpo, Republic of Korea). Serial dilutions of the homogenized samples were prepared in sterile 0.85% (*w*/*v*) NaCl. Each dilution was plated on bromocresol purple (BCP)-de Man-Rogosa-Sharpe (MRS; BD, Franklin Lakes, NJ, USA) agar. The composition of the MRS medium per liter included protease peptone No.3 (10.0 g), beef extract (10.0 g), yeast extract (5.0 g), glucose (20.0 g), sodium acetate (5.0 g), polysorbate 80 (1.0 g), dipotassium hydrogen phosphate (2.0 g), ammonium citrate (2.0 g), magnesium sulfate (0.1 g), and manganese sulfate (0.05 g), with water. The plates were incubated anaerobically at 37 °C for 20 h. Ten colonies displaying a yellow color on the BCP-MRS agar medium were selected from each sample and subcultured twice on the fresh BCP-MRS agar medium. The isolated strain was confirmed to be a Gram-positive and catalase-negative lactic acid bacterium via Gram staining and catalase testing.

### 2.2. Culture of Animal Cells

#### 2.2.1. Cell Lines and Culture Conditions

The murine macrophage cell line RAW 264.7 (Cat. No. KCLB40071), obtained from the Korean Cell Line Bank (Seoul, Republic of Korea), was cultured in Dulbecco’s modified Eagle’s medium (DMEM) purchased from Gibco (Grand Island, NY, USA). The culture medium was supplemented with 10% fetal bovine serum (FBS) and 1% penicillin-streptomycin solution (Gibco). The cells were maintained at 37 °C in a 5% CO_2_ atmosphere using a CO_2_ incubator from Thermo Fisher Scientific (Waltham, MA, USA). 

The human colon adenocarcinoma cell line Caco-2 (HTB-37™), obtained from the American Type Culture Collection (ATCC, Manassas, VA, USA), was cultured in DMEM supplemented with 10% fetal bovine serum (FBS) obtained from Corning Inc. (Corning, NY, USA) and 1% penicillin-streptomycin solution. The cells were incubated at 37 °C in a controlled environment with a 5% CO_2_ concentration. Cell detachment from the culture flask was achieved using 0.25% trypsin-ethylenediaminetetraacetic acid (0.25% trypsin-EDTA) purchased from Gibco.

#### 2.2.2. Preparation of the Lactic Acid Bacterial Sample

MRS broth was used as the growth medium and inoculated with a culture broth containing lactic acid bacteria at a final concentration of 1% (*v*/*v*). The mixture was incubated at 37 °C until the cell density of each lactic acid bacterial strain reached approximately 1 × 10^8^ CFU/mL. To isolate the bacterial cells, one milliliter of the culture broth was centrifuged five times at 16,000× *g* for 1 min each time. After each centrifugation step, the pellet was washed three times with Dulbecco’s phosphate-buffered saline (DPBS, Welgene, Gyeongsan, Republic of Korea). For subsequent experiments, the bacterial suspensions were resuspended and diluted in specific cell culture media according to the desired multiplicity of infection (MOI), which represents the ratio of viable bacterial cells to macrophages. In addition, bacterial suspensions were diluted in DMEM supplemented with 10% FBS to allow assessment of intestinal adhesion.

### 2.3. Identification of Lactic Acid Bacteria

#### 2.3.1. Nucleotide Sequencing of 16S rRNA Gene

For isolating genomic DNA from each strain, a single colony grown on MRS agar was inoculated into MRS broth and incubated at 37 °C for 16 h. After incubation, 3 mL of the lactic acid bacteria culture was harvested via centrifugation at 21,000× *g* for 1 min at 4 °C. The AccuPrep^®^ Genomic DNA Extraction Kit (BIONEER, Daejeon, Republic of Korea) was used to extract DNA from Gram-positive bacteria following the manufacturer’s protocol and using lysis buffer (20 mM Tris-HCl, pH 8.0, 2 mM EDTA, 1.2% Triton^®^ X-100) and lysozyme (60 mg/mL). Polymerase chain reaction (PCR) premix (AccuPower^®^ PCR PreMix, BIONEER) was used according to the manufacturer’s protocol for PCR amplification for species identification. PCR was performed using 20 ng template DNA, 27F primer (5′- AGA GTT TGA TCA TGG CTC AG -3′), and 1492R (5′- TAC GGY TAC CTT GTT ACG AC -3′). A thermocycler (TurboCycler; Blue-Ray Biotech, Taipei, Taiwan) was used under the conditions listed in Table 1.

After PCR amplification, DNA was purified using a QIAquick^®^ PCR purification kit (Qiagen, Hilden, Germany). The purified DNA was analyzed using a high-throughput DNA analyzer (3730XL; Thermo Fisher Scientific) installed at Bionics (Seoul, Republic of Korea). The obtained nucleotide sequences of the 16S rRNA gene were compared with available sequences using the Basic Local Alignment Search Tool (BLAST) provided by the National Center for Biotechnology Information (NCBI; National Institutes of Health, Bethesda, MD, USA (https://blast.ncbi.nlm.nih.gov/Blast.cgi, accessed on 6 May 2022). Phylogenetic trees were constructed to visualize the evolutionary relationships among different bacterial strains. Multiple sequence alignments of the 16S rRNA gene sequences were performed using ClustalX software version 2.1 [13]. Phylogenetic trees were generated using Molecular Evolutionary Genetics Analysis (MEGA) software version 11 [14], which employs algorithms such as neighbor-joining or maximum likelihood to infer evolutionary relationships.

#### 2.3.2. Scanning Electron Microscopy

The lactic acid bacterial strains were observed using a Hitachi H-7600 scanning electron microscope (SEM) at the Industry-Academia Cooperation Center, Eulji University (Seongnam, Republic of Korea). The bacterial sample was fixed with 2.5% glutaraldehyde for 2 h, followed by washing with 0.1 M phosphate buffer (pH 7.4) for 15 min. A secondary fixation step was performed using 1% osmium tetroxide solution for 1 h. After fixation, the samples were washed with the same buffer. Dehydration was performed in a series of ethyl alcohol solutions with 50–100% concentrations for 15 min each. The substitution was performed by treating the sample with a mixture of isoamyl acetate and 100% ethyl alcohol for 15 min at ratios of 1:2, 1:1, 2:1, and then with isoamyl acetate alone. The samples were dried using a Hitachi HCP-2 critical-point dryer with CO_2_ gas, mounted on a stub with carbon tape, and coated with platinum using a Hitachi E-1030 instrument. Finally, the coated stubs were observed using a Hitachi S-4700 SEM. The acquired SEM images were analyzed for size using ImageJ software version 1.8.0 [15]. 

#### 2.3.3. Carbohydrate Utilization Pattern of Lactic Acid Bacteria

The carbohydrate utilization pattern was determined using the API 50 CHL kit (API System, BioMérieux, Montalieu Vercie, France) according to the manufacturer’s instructions. This kit allows the identification of different carbohydrates that can be utilized by the tested bacterial strains. The API 50 CHL kit provides a series of microtubes containing various carbohydrate substrates. The growth and metabolic reactions of the bacterial strains in response to these substrates were assessed and recorded to obtain information regarding their carbohydrate utilization patterns.

### 2.4. Evaluation of the Probiotic Characteristics of Lactic Acid Bacteria

#### 2.4.1. Acid Tolerance 

A lactic acid bacterial culture was incubated at 37 °C for 20 h. The culture was added to the MRS broth at a final concentration of 1% (*v*/*v*) and incubated at 37 °C for 16 h. The culture broth was centrifuged at 21,000× *g* for 1 min, and the pellets were washed twice with 0.85% (*w*/*v*) NaCl. The washed pellets were reconstituted in 100 mM glycine-HCl buffer at pH 2.5. The bacterial suspension was then serially diluted with 0.85% (*w*/*v*) NaCl at the beginning (0 h) and plated on Petrifilm™ Lactic Acid Bacteria Count Plates (3M, Saint Paul, MN, USA) as control samples. After 2 h of incubation at 37 °C, the bacterial suspensions were serially diluted again with 0.85% (*w*/*v*) NaCl and plated on Petrifilm™ as experimental samples. The Petrifilm™ plates were then incubated at 37 °C for 20 h, and the viable cell count was determined. Acid tolerance was calculated using the following formula:

(1) $$Acid\;tolerance\left(\%\right)=\frac{\log\left(Viable\;cell\;number\;after\;2\;h\;\left(sample\right)\right)}{\log\left(Viable\;cell\;number\;after\;0\;h\;\left(control\right)\right)}\times 100\;(\%).$$

#### 2.4.2. Bile Tolerance 

Lactic acid bacteria were incubated in MRS broth at 37 °C for 20 h. The bacterial culture was added to supplement with or without 0.3% (*w*/*v*) Bacto-Oxgall medium (BD) to a final concentration of 0.5% (*v*/*v*). After 16 h of incubation at 37 °C, viable cell count was determined as described in Section 2.4.1. Bile tolerance was calculated using the following formula:

(2) $$Bile\;tolerance(\%)=\frac{log(Viable\;cell\;number\;in\;the\;presence\;of\;Oxgall)}{\log(Viable\;cell\;number\;in\;the\;absence\;of\;Oxgall)}\times 100\;(\%).$$

#### 2.4.3. Pancreatin Tolerance 

The preparation of bacterial cells was the same as described in Section 2.4.1. The washed bacterial pellets were reconstituted in MRS broth supplemented with 2% (*w*/*v*) sucrose and 0.5% pancreatin (Sigma-Aldrich, St. Louis, MO, USA). Viable cell count was determined as described in Section 2.4.1. Pancreatin tolerance was calculated using the following formula:

(3) $$Pancreatin\;tolerance\;(\%)=\frac{log(Viable\;cell\;number\;after\;2\;h\;(sample))}{\log(Viable\;cell\;number\;after\;0\;h\;(control))}\times 100\;(\%).$$

#### 2.4.4. Gut Adhesion Ability

Caco-2 cells were seeded at a density of 5 × 10^5^ cells per well in a 24-well plate. The plate was then incubated at 37 °C with 5% CO_2_ for 20 h. The cells were treated with lactic acid bacteria sample at a concentration of 5 × 10^7^ CFU/mL. The 24-well plate was incubated at 37 °C with 5% CO_2_ for 2 h to allow the cells to adhere. In parallel, control samples were prepared by incubating lactic acid bacteria (5 × 10^7^ CFU/mL) in antibiotic-free cell culture medium at 37 °C for 2 h. To remove non-adherent bacterial cells, the culture medium was aspirated, and the cell monolayers were washed four times with DPBS. A total of 200 μL of 0.25% trypsin-EDTA was added to each well, and the plate was incubated at 37 °C with 5% CO_2_ for 5 min. The cells were collected by transferring them to new tubes. They were then washed twice with DPBS via centrifugation at 21,000× *g* for 1 min each time. The cells were then treated with 0.1% (*v*/*v*) Triton X-100 in DPBS. Both experimental and control samples were plated on Petrifilm™ plates. The plates were then incubated at 37 °C for 16 h, and the number of viable cells was counted. Percent adherence was determined using the following formula:

(4) $$Gut\;adhesion\;ability\left(\%\right)=\frac{log(Viable\;cell\;number\;of\;sample)}{log(Viable\;cell\;number\;of\;control)}\times 100\;(\%).$$

#### 2.4.5. Antioxidant Activity

The antioxidant activity of the probiotic strains was evaluated using the DPPH radical scavenging assay. Briefly, 2,2-diphenyl-1-picrylhydrazyl (DPPH, Sigma-Aldrich) was used as a radical scavenger. A standard curve was constructed using 0.0625, 0.125, 0.25, 0.5, 1, and 2 mM ascorbic acid, with 2 mM ascorbic acid as positive control and distilled water as negative control.

The probiotic culture was prepared by inoculating the cultured broth, obtained by incubating the strains in MRS broth at 37 °C for 20 h, into fresh MRS broth to achieve a 1% (*v*/*v*) concentration. The culture was then incubated at 37 °C for 16 h. After incubation, the culture was centrifuged at 21,000× *g* for 5 min, and 50 μL of the culture supernatant or control solution was transferred to a 1.5 mL tube.

A DPPH solution (0.1 mM) was prepared by dissolving 2 mM DPPH in methanol and diluting the solution with methanol. To initiate the reaction, 950 μL of the 0.1 mM DPPH solution was added to the 1.5 mL tube containing the probiotic culture supernatant; the mixture was then allowed to react at 20 °C for 30 min. After the reaction, the mixture was centrifuged at 16,000× *g* for 1 min, and 200 μL of the supernatant was transferred to a 96-well plate. The absorbance was measured at 517 nm using a microplate reader (Epoch microplate reader, Biotek Instruments, Inc., Winooski, VT, USA).

DPPH radical scavenging activity was calculated using the following equation and expressed as a percentage compared to the DPPH radical scavenging activity of 2 mM ascorbic acid:

(5) $$DPPH\;radical\;scavenging\;activity\;(\%)=\left(1-\frac{A_{sample}}{A_{control}}\right)\times 100\;\left(\%\right),$$

where A_control_ is the absorbance of the negative control (distilled water), and A_sample_ is the absorbance of the probiotic culture supernatant or positive control solution.

### 2.5. Evaluation of Immunostimulatory Activities

#### 2.5.1. Cell Viability Assay

Cytotoxicity of the probiotic strain was assessed in RAW 264.7 cells. RAW 264.7 cells were seeded in a 96-well plate at a density of 5 × 10^4^ cells/well, with 100 μL of cell suspension per well, and incubated at 37 °C with 5% CO_2_ for 20 h. The negative control group was treated with MOI 0, while the probiotic samples at MOI 50, 100, and 200 were added to the respective wells at 100 μL per well, and the cells were incubated at 37 °C with 5% CO_2_ for 24 h. Cell cytotoxicity was measured using EZ-Cytox (DoGenBio Co., Ltd., Seoul, Republic of Korea). After incubation, the culture medium was removed, and 200 μL of DMEM containing 10% FBS and 1% P/S was added to each well, followed by adding 20 μL of EZ-Cytox. The plate was then incubated in a 5% CO_2_ incubator at 37 °C for 30 min. For blank measurements, the same procedure was performed in wells without cells. To measure the viability of RAW 264.7 cells, absorbance was measured at 450 nm using a microplate reader once the medium turned orange. The following formula was used for the calculation:

(6) $$Cell\;viability\;(\%)=(\frac{A_{sample}{-A}_{blank}}{A_{control}\;{-A}_{blank}})\times \;100(\%).$$

#### 2.5.2. Nitric Oxide Assay

NO production in RAW 264.7 macrophages was measured using the Griess reagent. The Griess reagent consists of two components: Griess reagent A and Griess reagent B. Griess reagent A contains 0.1% *N*-(1-naphthyl) ethylenediamine dihydrochloride (Sigma-Aldrich), whereas Griess reagent B contains 1% sulfanilamide (Sigma-Aldrich) in 5% phosphoric acid (Sigma-Aldrich). RAW 264.7 cells were seeded in a 96-well plate at a density of 5 × 10^5^ cells/mL, with 500 μL of cell suspension per well, and incubated at 37 °C with 5% CO_2_ for 20 h. The probiotic strain corresponding to an MOI of 100 (5 × 10^7^ CFU), the positive control group treated with lipopolysaccharides (LPS) (Cat. # L4391, Sigma-Aldrich), and the negative control group (MOI 0) were added to RAW 264.7 cells, and the cells were cultured for 24 h. After incubation, 100 μL of the culture supernatant was transferred to a new 96-well plate, and equal volumes of Griess reagents A and B were mixed and added to the wells. The plate was then incubated at 20 °C for 15 min, and absorbance was measured at 540 nm using a microplate reader. Standard curves for NO concentration were generated using serially diluted samples of NaNO_2_ ranging from 7.815 to 250 μM in DMEM, and the measured absorbance values were used to calculate the NO concentrations. The NO production in samples treated with lactic acid bacteria was compared to that in the positive control group treated with LPS, which was set as 100%.

#### 2.5.3. Total RNA Extraction 

RAW 264.7 cells were plated in a 6-well plate at a density of 1 × 10^6^ cells/well and incubated at 37 °C in a 5% CO_2_ environment for 20 h. The cells were exposed to the probiotic strain to achieve an MOI of 100. The positive control group was treated with 1 μg/mL LPS, whereas the negative control group (MOI = 0) was left untreated. All groups were incubated at 37 °C in a 5% CO_2_ atmosphere for 24 h. After incubation, the cells were washed twice with DPBS. To isolate the cells, the easy-BLUE™ Total RNA Extraction Kit (iNtRON Biotechnology, Inc., Seongnam, Republic of Korea) was used, and the harvested cells were transferred to a 1.5 mL tube. Each tube was then treated with 200 μL chloroform (Sigma-Aldrich) and vortexed for 10 s. After centrifugation at 16,000× *g* for 10 min at 4 °C, the upper aqueous phase, approximately 400 μL, was transferred to a fresh 1.5 mL tube. To induce RNA precipitation, 400 μL of 2-propanol (Sigma-Aldrich) was added, mixed, and incubated for 10 min at 20 °C. After a centrifugation phase at 16,000× *g* for 5 min at 4 °C, the supernatant was discarded, and the remaining RNA pellet was cleaned with 1 mL of 75% ethanol (Sigma-Aldrich). After another round of centrifugation at 10,000× *g* for 5 min at 4 °C, the RNA pellet was air dried to the extent possible and then solubilized in 30 μL RNA-grade DEPC-DW (BIONEER).

#### 2.5.4. Synthesis of cDNA and Real-Time Quantitative PCR (RT-qPCR)

Following the RNA extraction process, the concentration and purity of the extracted RNA were measured using Take3 Micro-Volume Plates (Epoch Microplate Reader, Biotek Instruments, Inc.) as an essential step in the synthesis of complementary DNA (cDNA). cDNA was synthesized using the CellScript™ cDNA Master Mix (Cellsafe, Suwon, Republic of Korea) on a PCR machine. The master mix for the reaction included the optimal ratios of oligo(dT) primers, MIMLV reverse transcriptase, dNTPs, and a ribonuclease inhibitor. The specific conditions for cDNA synthesis are listed in Table 1. RT-qPCR was performed using QGreenBlue 2× qPCR Master Mix (CellSafe) according to the manufacturer’s guidelines. The reaction mix contained 500 ng of cDNA and the appropriate primers. Amplification was performed on a QuantStudio™ 1 Real-Time PCR System (Thermo Fisher Scientific). The primer sequences are shown in Table 1. RT-qPCR was performed according to the manufacturer’s protocol. Glyceraldehyde-3-phosphate dehydrogenase (GAPDH) was used as a reference gene for normalization, and relative expression levels of cytokine mRNA were calculated using the ΔΔCT method [16]. 

#### 2.5.5. Protein Extraction

After applying the probiotic strain to RAW 264.7 cells, the cells were rinsed twice with DPBS (Welgene) according to the procedure used for RNA isolation. The cells were then incubated for 30 min at 0 °C with 130 μL radioimmunoprecipitation assay buffer (RIPA buffer, Cell Signaling Technology, Beverly, MA, USA) containing 1% (*v*/*v*) protease inhibitor cocktail (GenDEPOT, Katy, TX, USA) and 1% (*v*/*v*) phosphatase inhibitor cocktail (GenDEPOT). The cells were collected using a cell scraper from each well, transferred to a 1.5 mL tube, and vortexed for 10 s. This procedure was repeated thrice. After a subsequent incubation period of 2 h at 0 °C, cell lysates were centrifuged at 27,000× *g* for 10 min at 4 °C, after which the supernatant was transferred to a fresh 1.5 mL tube for protein extraction. Finally, the concentration of the isolated protein was determined using the Pierce™ BCA Protein Assay Kit (Thermo Fisher Scientific).

#### 2.5.6. Western Blotting

In total, 30 µg of the isolated protein was separated via 12.5% SDS-PAGE. Western blotting was performed by transferring proteins from the gel to a nitrocellulose membrane (Bio-Rad Laboratories, Inc., Hercules, CA, USA) using a TE70X Semi-Dry Transfer Unit (Hoefer Inc., Holliston, MA, USA) at 45 mV for 2 h. The membrane was then subjected to blocking with a buffer containing 3% (*w*/*v*) bovine serum albumin fraction V (BSA, Roche, Basel, Switzerland) in Tris-buffered saline with 0.5% (*v*/*v*) Tween-20 (TBS-T, iNtRON Biotechnology, Inc.), gently agitated for 30 min at 20 °C. The membrane was then incubated with primary antibodies at a 1:1000 dilution in TBS-T containing 3% BSA at 20 °C under gentle shaking for 1 h. The primary antibodies used included P38, phospho-P38, extracellular signal-regulated kinase (ERK), phospho-ERK, c-Jun N-terminal kinases (JNK), phospho-JNK, and β-actin (Cell Signaling Technology). After the primary antibody incubation was completed, the membrane was rinsed four times with 6 mL of TBS-T at 15 min intervals. The membrane was then incubated with a secondary antibody (anti-rabbit IgG, Cell Signaling Technology) at a 1:1000 dilution in TBS-T containing 3% BSA at 20 °C, again with gentle shaking, for 1 h. After secondary antibody incubation, the membrane was washed four times with 6 mL of TBS-T at 15 min intervals. Finally, protein bands were detected using an ECL solution (EzWestLumi plus, ATTO Corporation, Tokyo, Japan), and images were captured using the Amersham™ Imager 600 (Cytiva, Amersham, UK). Quantification of protein bands was performed using ImageJ software (National Institutes of Health, Bethesda, MD, USA) with β-actin as the reference protein for Western blotting analysis [15]. The quantified protein expression was then compared to the negative control group, which was set as 100%. 

### 2.6. Statistical Analyses

Experiments were performed in triplicate, with each run consisting of three replicates. Results are expressed as mean ± standard deviation. Statistical analysis was performed with IBM SPSS Statistics version 26.0 (IBM Corp, Armonk, NY, USA). Significant differences between groups were determined using independent samples *t*-tests and one-way analysis of variance. To ensure the validity of the ANOVA analysis, the Shapiro–Wilk test was used to assess the normality of the residuals, and the Levene test was used to assess the homogeneity of variances. Duncan’s multiple range test was used for further evaluation. All statistical analyses were performed at a significance level of *p* < 0.05.

## 3. Results

### 3.1. Selection of Lactic Acid Bacteria with Immunostimulatory Function and Identification of the Selected Strain

#### 3.1.1. Selection of Lactic Acid Bacteria with Immunostimulatory Function 

In total, 58 lactic acid bacterial strains were isolated from eight types of plants from Jeju Island. Through 16S rRNA gene sequencing analysis, six strains, namely *Lactococcus lactis* subsp. *lactis* CAB1001, CAB401, CAB701, CAB702, CAB801, and CAB802, were found to belong to the probiotic species designated as health-functional foods by the Ministry of Food and Drug Safety in the Republic of Korea. All six strains exhibited high levels of NO production, with *L. lactis* subsp. *lactis* CAB701, showing the highest NO production. Therefore, *L. lactis* subsp. *lactis* CAB701 was selected for further analysis. 

#### 3.1.2. Identification of Selected Lactic Acid Bacteria Strain

**Figure 1:**
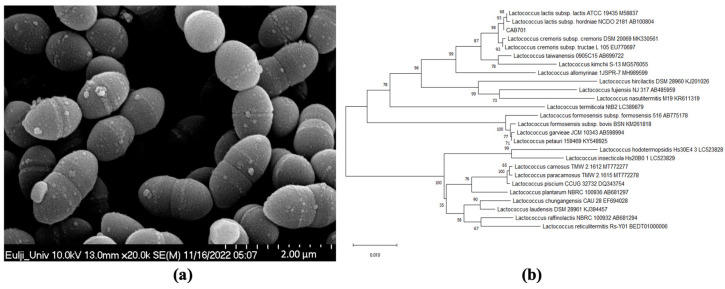
(**a**) Scanning electron microscopy image of *L. lactis* subsp. *lactis* CAB701, the scale bar represents 2 µm (×20,000). (**b**) Phylogenetic tree of *L. lactis* subsp. *lactis* CAB701.

The selected strain was identified as *L. lactis* subsp. *lactis* using phylogenetic tree analysis; SEM analysis showed that the length of the cells ranged from 1.17 to 1.821 µm, while the width ranged from 0.78 to 0.84 µm. The shape of the strain was spherical (Figure 1).

The carbohydrate utilization pattern of *L. lactis* subsp. *lactis* CAB701 confirmed that *L. lactis* subsp. *lactis* CAB701 can ferment various carbohydrates, as shown in Table 1. *L. lactis* subsp. *lactis* CAB701 could ferment l-arabinose, d-ribose, d-xylose, d-galactose, d-glucose, d-fructose, d-mannose, d-mannitol, *N*-acethylglucosamine, amygdalin, arbutin, esculin ferric citrate, salicin, d-cellobiose, d-maltose, d-lactose (bovine origin), d-saccharose, d-trehalose, inulin, amidon (starch), gentiobiose, and potassium gluconate (Table 2).

### 3.2. Probiotic Characteristics 

#### 3.2.1. Acid, Bile, and Pancreatin Tolerances and the Ability to Adhere to Caco-2 Cells

In terms of acid tolerance, *L. lactis* subsp. *lactis* CAB701 displayed a value of 47.6%, while *Lacticaseibacillus rhamnosus* GG(LGG) exhibited a higher acid tolerance at 83.3% (Table 3). Regarding bile tolerance, *L. lactis* subsp. *lactis* CAB701 showed a value of 63.6%, whereas LGG demonstrated higher tolerance at 88.2%. *L. lactis* subsp. *lactis* CAB701 exhibited a pancreatin tolerance of 101.9%, and LGG showed a slightly higher tolerance at 104.1%. In terms of the adhesion ability to gut epithelial cells, *L. lactis* subsp. *lactis* CAB701 demonstrated an adhesion rate of 75.2%, while LGG exhibited an adhesion rate of 77.2%.

#### 3.2.2. Antioxidant Activities of Lactic Acid Bacteria 

The DPPH radical scavenging activity of the test strain was evaluated and compared with the antioxidant activity of 2 mM ascorbic acid used as a 100% standard. *L. lactis* subsp. *lactis* CAB701 and LGG exhibited antioxidant activities of 95.6 and 97.2%, respectively, indicating their effective ability to scavenge DPPH radicals (Table 4).

### 3.3. Immunostimulatory Activities of L. lactis subsp. lactis CAB701

#### 3.3.1. Cell Viability

**Figure 2:**
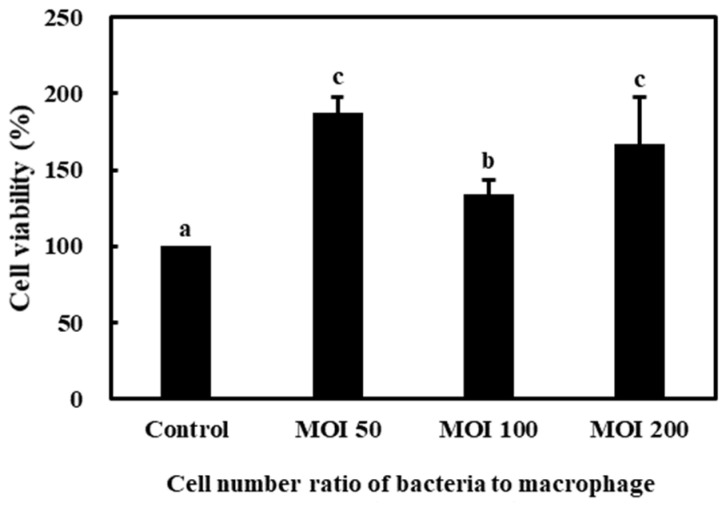
Effects of *L. lactis* subsp. *lactis* strains on the viability of RAW 264.7 cells. The multiplicity of infection (MOI, bacterial cell number/macrophage cell number) was 50, 100, and 200. Different letters among groups represent significant differences at *p* < 0.05.

The effects of *L. lactis* subsp. *lactis* CAB701 on the viability of RAW 264.7 cells are shown in Figure 2. The viability of RAW 264.7 cells treated with the *L. lactis* subsp. *lactis* CAB701 strain was more than 100% at all MOI, indicating that the *L. lactis* subsp. *lactis* CAB701 had no cytotoxic effect on RAW 264.7 cells.

#### 3.3.2. Production of NO

The stimulation of NO production using two bacterial strains, *L. lactis* subsp. *lactis* CAB701 and LGG, were evaluated in RAW 264.7 cells. The results demonstrated significant differences in NO production induced via the two strains. *L. lactis* subsp. *lactis* CAB701 exhibited a notably higher level of NO production at 86.3%, whereas LGG showed significantly lower NO production at 18.5% (Table 5). 

#### 3.3.3. Quantitative Analysis of Cytokine Expression in Macrophages Treated with Lactic Acid Bacteria

This study aimed to evaluate the immunomodulatory potential of *L. lactis* subsp. *lactis* CAB701 by comparing its effects with those of LPS, which was a positive control. Various inflammatory mediators, such as iNOS, TNF-α, COX-2, IL-1β, and IL-6, were examined to assess the immune response. In the LPS-treated group, significant upregulation was observed in the expression levels of iNOS, TNF-α, COX-2, IL-1β, and IL-6 compared to those in the control group, indicating a robust immune response. Interestingly, *L. lactis* subsp. *lactis* CAB701 also demonstrated notable effects on enhancing the expression of these immune mediators, albeit to a lesser extent than those with LPS.

**Figure 3:**
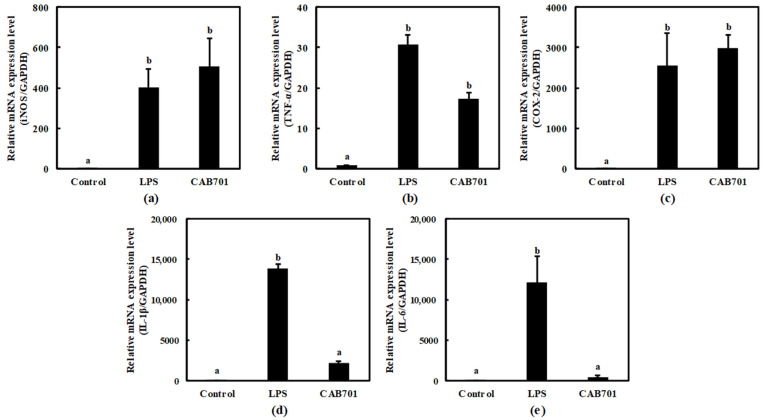
Effects of *L. lactis* subsp. *Lactis* CAB701 on the mRNA expression levels of (**a**) inducible nitric oxide synthase (*iNOS*), (**b**) tissue necrosis factor-α (*TNF-α*), (**c**) cyclooxygenase-2 (*COX-2*), (**d**) interleukin-1β (*IL-1β*), and (**e**) interleukin-6 (*IL-6*) genes in RAW 264.7 cells. The multiplicity of infection (MOI, bacterial cell number/macrophage cell number) was 100. Macrophages treated with 1 μg/mL of lipopolysaccharide (LPS) were used as the positive control sample. Different letters among groups represent significant differences at *p* < 0.05.

Notably, treatment with *L. lactis* subsp. *Lactis* CAB701 increased inducible nitric oxide synthase (iNOS) expression, highlighting its potential to stimulate the immune system. The expression levels of TNF-α and COX-2 were also significantly elevated upon *L. lactis* subsp. *Lactis* CAB701 treatment, highlighting its strong immunomodulatory effects (Figure 3a–c). Moreover, *L. lactis* subsp. *Lactis* CAB701 treatment led to a considerable increase in IL-1β expression, indicating its ability to activate immune responses. *L. lactis* subsp. *Lactis* CAB701 treatment also significantly upregulated IL-6 expression, suggesting its involvement in immune modulation (Figure 3d,e).

These findings emphasize the significant immunomodulatory potential of the CAB701 strain, although the expression levels of IL-1β and IL-6 did not surpass those induced via LPS.

#### 3.3.4. Quantitative Analysis of Mitogen-Activated Protein Kinase (MAPK) Pathway Protein Expression in Macrophages Treated with *L. lactis* subsp. *lactis* CAB701

**Figure 4:**
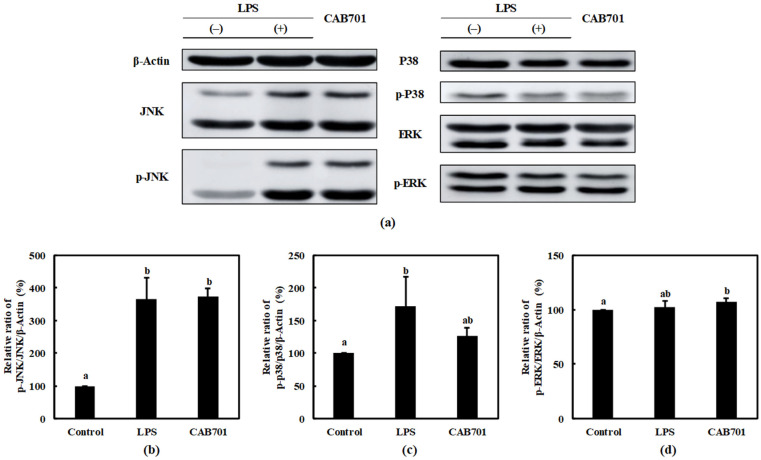
Effects of *L. lactis* subsp. *lactis* CAB701 treatment on the mitogen-activated protein kinase (MAPK) signaling pathway in RAW 264.7 macrophages. The multiplicity of infection (MOI, bacterial cell number/macrophage cell number) was 100. (**a**) Western blot reveals the expression of MAPK pathway proteins in macrophages treated with *L. lactis* subsp. *lactis* CAB701. Graphs depict the relative ratios of (**b**) p-JNK/JNK, (**c**) p-p38/p38, and (**d**) p-ERK/ERK in the treated macrophages. Western blots are representative of three independent experiments. Macrophages treated with 1 μg/mL of lipopolysaccharides (LPS) were used as the positive control sample. Different letters among groups represent significant differences at *p* < 0.05.

An increased p-JNK/JNK expression was observed in the *L. lactis* subsp. *lactis* CAB701-treated group compared to that in the control group. Notably, the expression level of p-JNK/JNK in the *L. lactis* subsp. *lactis* CAB701-treated group was similar to that in the LPS-treated group. *L. lactis* subsp. *lactis* CAB701 treatment is suggested to activate the JNK pathway in RAW 264.7 cells to a similar extent as treatment with LPS (Figure 4b).

The expression of p-P38/P38 in response to *L. lactis* subsp. *lactis* CAB701 and LPS treatments were examined. Both the CAB701 and LPS treatment groups showed increased levels of p-P38/P38 compared to that in the control group (Figure 4c). Although LPS treatment significantly increased the expression of p-P38/P38, no statistically significant increase was observed in the *L. lactis* subsp. *lactis* CAB701-treated group. Nevertheless, the expression of p-P38/P38 in the *L. lactis* subsp. *lactis* CAB701-treated group was higher than that in the control group, indicating that *L. lactis* subsp. *lactis* CAB701 treatment modulated P38 pathway activation. 

The expression of p-ERK/ERK was assessed in response to *L. lactis* subsp. *lactis* CAB701 and LPS treatments. No statistically significant difference in p-ERK/ERK expression was observed between LPS-treated and control groups. However, the *L. lactis* subsp. *lactis* CAB701-treated group exhibited a slightly higher expression of p-ERK/ERK, suggesting that *L. lactis* subsp. *lactis* CAB701 may play a role in modulating the activation of the ERK pathway (Figure 4d).

## 4. Discussion

A potential probiotic strain must exhibit superior characteristics to exert its beneficial effects. Desirable probiotic properties include the ability of the strain to withstand the acidic and bile conditions of the digestive system, adherence to mucosal surfaces, antibacterial activity against pathogens, and retention of bile acid hydrolase activity [17].

The lactic acid bacteria isolated in this study were identified as *Lactococcus lactis* subsp. *lactis* via phylogenetic tree analysis based on the nucleotide sequence of the 16S rRNA gene named CAB701, and the identification of this strain at the level of subspecies was validated via whole genome sequence analysis.

*Lactococcus lactis* subsp. *lactis* CAB701 exhibited exceptional resilience under acidic conditions, demonstrating a survival rate of 47.57% even after 2 h of treatment at pH 2.5. The ability of CAB701 to withstand low pH conditions for extended periods suggests its potential application in the production of acidic foods and beverages, where the survival and function of microbes are essential. This present study highlights a contrasting behavior compared to that of previously identified *Lactococcus lactis* strains such as NABRII49, NABRII66, NABRII64, NABRII61, and NABRII67, all of which were completely eliminated after 1.5 h of treatment at pH 2.5 [18].

*L. lactis* subsp. *lactis* CAB701 showed a bile tolerance of 63.6%, indicating its survivability against the detrimental effects of bile salts. This ability is crucial for probiotic bacteria to maintain their viability and functionality while traversing the digestive system. *L. lactis* subsp. *lactis* CAB701 demonstrated a pancreatin tolerance of 101.9%, highlighting its ability to withstand the activity of pancreatic enzymes, which is essential for its survival and activity in the small intestine, where pancreatic enzymes play a significant role in digestion. Regarding adhesion ability, *L. lactis* subsp. *lactis* CAB701 exhibited a remarkable adhesion rate of 75.2% to Caco-2 cells, indicating a strong potential for the strain to establish beneficial interactions with intestinal epithelial cells, which is crucial for exerting probiotic effects and modulating host health. Overall, *L. lactis* subsp. *lactis* CAB701 demonstrates acid tolerance, bile tolerance, pancreatin tolerance, and adhesion. These characteristics make it a promising candidate for further investigation and potential applications as a probiotic strain. Although the acid tolerance, bile tolerance, and pancreatin tolerance of CAB701 are sufficiently characterized to be used as a probiotic strain, their characteristics are lower than those of LGG, a representative strain used commercially, so encapsulation of the strain may be considered to increase the commercial availability of CAB701.

Upon oral ingestion, probiotics undergo a challenging journey through the digestive tract, where these microorganisms must first survive the harsh conditions in the stomach and small intestine, including low pH (highly acidic environment), bile salts, and pancreatic enzymes [19]. Survival is paramount as it dictates the ability of probiotics to reach their primary site of action, the large intestine, in sufficient numbers. The stomach is the first major obstacle for orally ingested probiotics as its highly acidic environment (pH approximately 2) is lethal to many bacteria. However, effective probiotic strains can survive this hostile environment primarily because of their acid tolerance mechanisms. These include the production of stress proteins, changes in membrane fatty acid composition, and the ability to pump protons out of cells [20]. As probiotics move into the small intestine, they encounter bile, a digestive fluid produced by the liver and stored in the gallbladder. Bile contains bile salts, which are detergent-like substances that damage bacterial cell membranes and cause cell death. Probiotics must be bile-tolerant to colonize the small intestine and move to the large intestine. The mechanisms of bile tolerance in probiotics include the expressions of bile salt hydrolase, an enzyme that modifies and inactivates bile salts, and efflux pumps that remove harmful bile salts from cells [21]. Pancreatin, a mixture of digestive enzymes produced by the pancreas, is released into the small intestine to aid digestion. Probiotics must be resistant to these enzymes to maintain their viability and function. This resistance to enzyme action can be conferred via the production of protective proteins or changes in the cell wall structure [22].

The adhesion of probiotics to the GI tract lining is a crucial determinant of their efficacy. Beneficial bacteria must adhere to the intestinal mucosa to effectively colonize the gut and provide health benefits. By adhering to the intestinal lining, probiotics can establish a foothold in the gut, resist washing during digestion, and interact more effectively with the host immune system. The adhesion process involves specific interactions between probiotics and the mucosal surfaces of the GI tract. Numerous factors influence such interactions, including the properties of the bacterial cell surface, characteristics of the intestinal mucosa, and overall environmental conditions within the GI tract [23]. Strongly adherent probiotics exert beneficial effects through several mechanisms. First, they can prevent harmful microorganisms from establishing a presence by occupying space and resources on the intestinal lining [24]. Second, adherent probiotics are positioned favorably to interact with the host immune system, potentially stimulating immune responses and improving the host’s infection resistance [25]. Probiotic adhesion can also reinforce gut barrier function, reducing the risk of harmful substances entering the bloodstream from the intestine [26]. Some studies have suggested that adherent probiotics can modulate tight junctions between intestinal epithelial cells, enhance the intestinal barrier, and reduce the effect of harmful bacteria and their toxins on the body [27]. Moreover, adhesion enables prolonged residence of probiotics in the GI tract, potentially amplifying their beneficial effects [28]. 

*L. lactis* subsp. *lactis* CAB701, LGG, and 2 mM ascorbic acid used as positive controls showed no significant effect on antioxidant activity, suggesting they have significant antioxidant potential. The ability to scavenge DPPH radicals indicates the ability of an organism to neutralize harmful free radicals that can cause oxidative damage in the body. Antioxidants play crucial roles in maintaining cellular health and protecting cells against various diseases associated with oxidative stress [29]. The significant antioxidant potential of these probiotic strains may have important implications for human health. Antioxidants help reduce oxidative stress and inflammation, which are the underlying factors in the development of several chronic diseases, such as cardiovascular disease, cancer, and neurodegenerative disorders [30]. LGG demonstrated antioxidant activity by effectively inhibiting the H_2_O_2_ generation induced via ferrous ions. Preclinical studies have provided evidence of the ability of LGG to reduce chronic inflammation associated with cancer development, further emphasizing its role in combating oxidative stress and related pathologies [31]. Considering these findings, *L. lactis* subsp. *lactis* CAB701 can be considered to possess unique antioxidant properties similar to those of LGG. This suggests that *L. lactis* subsp. *lactis* CAB701 reduces oxidative stress and inflammation, which are critical for maintaining overall health and preventing chronic diseases.

SEM analysis of the strain identified as *L. lactis* subsp. *lactis* CAB701 revealed the presence of cocci arranged in pairs and exhibited short-chain formation. This observation is consistent with the results obtained for *L. lactis* subsp. *lactis* Lac3 isolated from traditional fermented buffalo milk and the bacteriocin-producing probiotic strain *L. lactis* CH3 [32,33]. Extensive genomic analyses of lactococci suggest that plants are a significant source of dairy strains, including *L. lactis* subsp. *lactis* [34]. In line with these findings, the strain *L. lactis* subsp. *lactis* CAB701 was successfully isolated from cabbage originating on Jeju Island, reinforcing the hypothesis of the plant-based origins of this bacterial species and demonstrating its potential for discovering new probiotic candidates in diverse plant environments. 

According to the results obtained using API 50 CHL, *L. lactis* subsp. *lactis* CAB701 can utilize l-arabinose, d-ribose, d-xylose, d-galactose, d-glucose, d-fructose, d-mannose, d-mannitol, *N*-acetylglucosamine, amygdalin, arbutin, esculin ferric citrate, salicin, d-cellobiose, d-maltose, d-lactose, d-sucrose, d-trehalose, inulin, amidon, gentiobiose, and potassium gluconate. *L. lactis* subsp. *lactis* CAB701 demonstrated a high similarity of 96% to the type-strain of *L. lactis* subsp. *lactis*. Notably, *L. lactis* subsp. *lactis* CAB701 has the biochemical property of fermenting inulin. The influence of prebiotic components on growth dynamics and bacteriocin production has been observed in *L. lactis* subsp. *lactis* CECT 4434 [35]. The results showed that adding inulin to the culture medium increased biomass production and reduced the production time of probiotic strains. Therefore, based on its biochemical characteristics, *L. lactis* subsp. *lactis* CAB701 may play a role in increasing the maximum specific growth rate using inulin as a carbon source. 

Studies have shown that *L. lactis* subsp. *lactis* CAB701 can modulate immune responses. Specifically, this strain stimulates the production of NO, an important immune signaling molecule. In the experimental setup, treatment of macrophages with *L. lactis* subsp. *lactis* CAB701 resulted in a high level of NO production, reaching 86.3%, as indicated in Table 5. The induction of NO via *L. lactis* subsp. *Lactis* CAB701 indicates its potential to regulate various immune processes. NO plays diverse roles in the immune system, including antimicrobial activity, regulation of inflammation, and modulation of immune cell function. By stimulating NO production, *L. lactis* subsp. *Lactis* CAB701 may enhance immune responses and potentially aid in combating infections or regulating immune-related disorders.

The objective of this present study was to investigate the immunomodulatory potential of *L. lactis* subsp. *Lactis* CAB701 by comparing its effects with lipopolysaccharide (LPS) as a positive control. The expression levels of various inflammatory mediators, including *iNOS*, *TNF-α*, *COX-2*, *IL-1β*, and *IL-6*, were examined to evaluate the immune response. The results demonstrated the significant upregulation of *iNOS*, *TNF-α*, *COX-2*, *IL-1β*, and *IL-6* gene expressions in the LPS-treated group compared to that in the control group, indicating a robust immune response. Interestingly, *L. lactis* subsp. *lactis* CAB701 also notably enhanced the expression of these immune mediators, albeit to a lesser extent than that with LPS. Treatment with *L. lactis* subsp. *lactis* CAB701 significantly increased *iNOS* gene expression, highlighting its potential to stimulate the immune system. The expression levels of *TNF-α* and *COX-2* were also significantly elevated upon *L. lactis* subsp. *lactis* CAB701 treatment, indicating strong immunomodulatory effects. Furthermore, *L. lactis* subsp. *lactis* CAB701 treatment considerably increased the *IL-1β* gene expression, suggesting its ability to activate the immune response. However, it did not reach the levels observed in the LPS-treated group and treatment with *L. lactis* subsp. *lactis* CAB701 led to significant upregulation of *IL-6* gene expression, implying its involvement in immune modulation. These findings emphasize the significant immunomodulatory potential of *L. lactis* subsp. *lactis* CAB701, even though the expression levels of *IL-1β* and *IL-6* genes did not surpass those induced via LPS. This suggests that *L. lactis* subsp. *lactis* CAB701 stimulates the immune response by influencing the expression of key inflammatory mediators.

**Figure 5:**
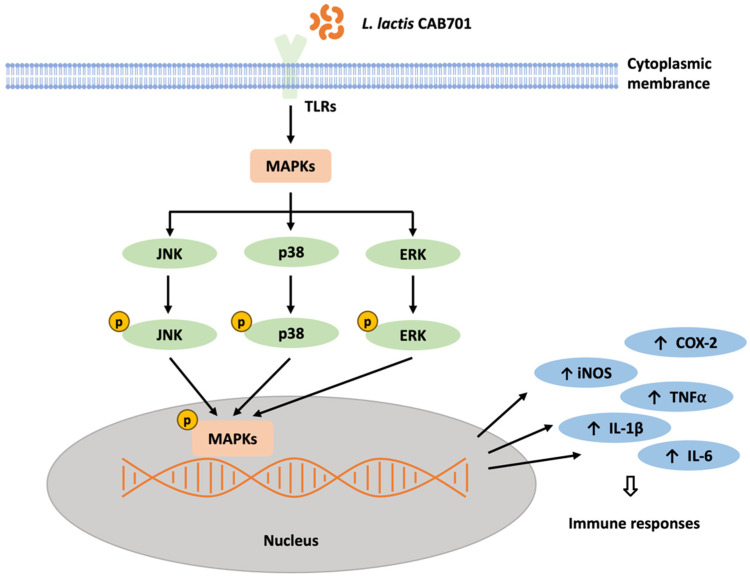
Schematic diagram depicting the predicted immunostimulatory signaling mechanism of *L. lactis* subsp. *lactis* CAB701 in RAW 264.7 macrophages. TLRs, Toll-like receptors; MAPKs, mitogen-activated protein kinases; JNK, c-Jun *N*-terminal kinases; ERK, extracellular signal-regulated kinase; iNOS, inducible nitric oxide synthase; TNF-α, tumor necrosis factor-alpha; IL-1β, interleukin -1 beta; IL-6, interleukin-6; COX-2, cyclooxygenase-2.

This study also investigated the effect of *L. lactis* subsp. *lactis* CAB701 on the activation of signaling proteins involved in the JNK, P38, and ERK pathways. The results revealed increased p-JNK/JNK expression in the *L. lactis* subsp. *lactis* CAB701-treated group, similar to that observed in the LPS-treated group. This suggests that *L. lactis* subsp. *lactis* CAB701 treatment activates the JNK pathway to a similar extent as that with LPS treatment, indicating its involvement in immune signaling. Regarding the P38 pathway, both *L. lactis* subsp. *lactis* CAB701 and LPS treatments led to increased levels of p-P38/P38 compared with those in the control group. Although LPS treatment significantly increased p-P38/P38 expression, no significant increase was observed in the *L. lactis* subsp. *lactis* CAB701-treated group. However, the expression of p-P38/P38 in the *L. lactis* subsp. *lactis* CAB701-treated group was higher than that in the control group, indicating that *L. lactis* subsp. *lactis* CAB701 treatment modulates activation of the P38 pathway. In the ERK pathway, no significant difference in p-ERK/ERK expression was observed between the LPS-treated and control groups. However, *L. lactis* subsp. *lactis* CAB701-treated group exhibited a slightly higher expression of p-ERK/ERK, suggesting that *L. lactis* subsp. *lactis* CAB701 may modulate ERK pathway activation. Overall, these results suggest that *L. lactis* subsp. *lactis* CAB701 possesses immunostimulatory potential by influencing the expression of key inflammatory mediators and activating the signaling pathways involved in immune responses (Figure 5). These findings contribute to understanding the mechanisms by which *L. lactis* subsp. *lactis* CAB701 stimulates the immune system and highlights its potential as a beneficial probiotic for immune-related applications. Further studies are required to explore the clinical implications of CAB701 in immune-related disorders.

## 5. Conclusions

These findings demonstrate the promising probiotic properties of *L. lactis* subsp. *lactis* CAB701. This strain exhibited a significant tolerance to acids, bile, and pancreatin, indicating its potential to survive under harsh conditions in the GI tract. It also displayed remarkable adhesion to Caco-2 cells, suggesting a strong potential for beneficial interactions with the intestinal epithelium. In addition, CAB701 exhibited antioxidant potential and strong immunostimulatory effects, which may contribute to the overall health benefits of this strain. Despite some differences compared to LGG, *L. lactis* subsp. *lactis* CAB701 shows promise as a probiotic strain. Notably, each probiotic strain has unique properties, and the observed differences may contribute to different health benefits. Overall, the results of this study provide valuable insights into the probiotic potential of *L. lactis* subsp. *lactis* CAB701. However, further studies are necessary to evaluate the efficacy of this strain using in vivo models and clinical trials.

## Figures and Tables

**Table 1:** Primer sequences for real-time quantitative polymerase chain reaction (RT-qPCR).

Gene		Primer Sequences	Size (bp)
*GAPDH*	F	5′- CAT GGC CTT CCG TGT TCC TAC -3′	122
	R	5′- TCA GTG GGC CCT CAG ATG C -3′	
*COX-2*	F	5′- CTC AGC CAT ACA GCA AAT CCT T -3′	101
	R	5′- GTC CGG GTA CAA TCG CAC TTA T -3′	
*iNOS*	F	5′- CCA GCC TGC CCC TTC AAT -3′	104
	R	5′- ATC CTT CGG CCC ACT TCC T -3′	
*IL-1β*	F	5′- TGA CGG ACC CCA AAA GAT -3′	122
	R	5′- GTG ATA CTG CCT GCC TGA AG -3′	
*IL-6*	F	5′- CCG GAG AGG AGA CTT CAC AGA G -3′	107
	R	5′- TCA TTT CCA CGA TTT CCC AGA G -3′	
*TNF-α*	F	5′- AGG CAC TCC CCC AAA AGA TG -3′	122
	R	5′- CAC CCC GAA GTT CAG TAG ACA GA -3′	

Abbreviations: *GAPDH*, glyceraldehyde-3-phosphate dehydrogenase; *COX-2*, cyclooxygenase-2; *iNOS*, inducible nitric oxide synthase; *IL-1β*, interleukin-1β; *IL-6*, interleukin-6 and *TNF-α*, tissue necrosis factor-α.

**Table 2:** Carbohydrate utilization of *L. lactis* subsp. *lactis* ATCC 19435 and *L. lactis* subsp. *lactis* CAB701.

Ingredients	*L. lactis* subsp. *lactis* NCTC 6681 *	*L. lactis* subsp. *lactis* CAB701	Ingredients	*L. lactis* subsp. *lactis* NCTC 6681	*L. lactis* subsp. *lactis* CAB701
Glycerol	− **	−	Esculin ferric citrate	+	+
Erythritol	−	−	Salicin	+	+
d-Arabinose	−	−	d-Cellobiose	+	+
l-Arabinose	−	−	d-Maltose	+	+
d-Ribose	+	+	d-Lactose (bovine origin)	+	+
d-Xylose	+	+	d-Melibiose	−	−
l-Xylose	+	+	d-Saccharose (sucrose)	+	+
d-Adonitol	−	−	d-Trehalose	+	+
Methyl-β-d- xylopyranoside	−	−	Inulin	−	+
d-Galactose	−	−	d-Melezitose	−	−
d-Glucose	+	+	d-Raffinose	−	−
d-Fructose	+	+	Amidon (starch)	+	+
d-Mannose	+	+	Glycogen	−	−
l-Sorbose	+	+	Xylitol	−	−
l-Rhanmose	−	−	Gentiobiose	+	+
Dulcitol	−	−	d-Turanose	−	−
Inositol	−	−	d-Lyxose	−	−
d-Mannitol	−	−	d-Tagatose	−	−
d-Sorbitol	+	+	d-Fucose	−	−
Methyl-α-d- mannopyranoside	−	−	l-Fucose	−	−
Methyl-α-d- glucopyranoside	−	−	d-Arabitol	−	−
*N*-Acetyl glucosamine	−	−	l-Arabitol	−	−
Amygdalin	+	+	Potassium Gluconate	+	+
Arbutin	+	+	Potassium 2- ketogluconate	−	−
Esculin ferric citrate	+	+	Potassium 5- ketogluconate	−	−

* *L. lactis* subsp. *lactis* strain NCTC 6681 is a bacterial type-strain ** +: Positive reaction; −: Negative reaction.

**Table 3:** Gastrointestinal tract tolerance and gut adhesion ability of *L. lactis* subsp. *lactis* CAB701.

Strain	Acid-Tolerance (%)	Bile-Tolerance (%)	Pancreatin- Tolerance (%)	Adhesion Ability (%)
*Lactococcus lactis* subsp. *lactis* CAB701	* 47.6 ± 2.0	* 63.6 ± 9.3	* 101.9 ± 0.3	75.2 ± 5.4
*Lacticaseibacillus rhamnosus* GG	83.3 ± 2.3	88.2 ± 0.8	104.1 ± 0.3	77.2 ± 1.4

Data are expressed as mean ± standard deviation of three independent experiments. Significant differences are denoted by asterisks (*, *p* < 0.05).

**Table 4:** DPPH radical scavenging activities.

Strain	Antioxidant Activity (%)
*Lactococcus lactis* subsp. *lactis* CAB701	95.6 ± 1.3
*Lacticaseibacillus rhamnosus* GG	97.2 ± 0.6

Data are expressed as mean ± standard deviation of three independent experiments. No significant difference was observed between the groups.

**Table 5:** Nitric oxide (NO) production by RAW 264.7 macrophages treated with *L. lactis* subsp. *lactis* CAB701. The multiplicity of infection (MOI, bacterial cell number/macrophage cell number) was 100.

Strain	NO Production (%)
*Lactococcus lactis* subsp. *lactis* CAB701	* 86.3 ± 6.8
*Lacticaseibacillus rhamnosus* GG	18.5 ± 3.2

Data are expressed as the mean ± standard deviation of three independent experiments. A significant difference is indicated by an asterisk (*, *p* < 0.05).

## Data Availability

Not applicable.
